# Potential Role of Sphingosine-1-Phosphate and Its Receptors in the Reparative Processes of the Adult Brain

**DOI:** 10.3390/ijms27167342

**Published:** 2026-08-17

**Authors:** Uliana A. Gutner, Maria A. Shupik, Alice Alessenko

**Affiliations:** Emanuel Institute of Biochemical Physics of the Russian Academy of Sciences, Moscow 119334, Russia; mariashupik@gmail.com (M.A.S.); alicealessenko@gmail.com (A.A.)

**Keywords:** sphingosine-1-phosphate, sphingosine-1-phosphate receptors, neurogenesis, neurogenic niche, neurotrophins, oligodendrogenesis, angiogenesis

## Abstract

This review highlights the main aspects of the involvement of sphingosine-1-phosphate (S1P) metabolism in the reparative processes of the adult brain, including neurogenesis, oligodendrogenesis and angiogenesis, which are necessary for the normal functioning of the central nervous system. Neurogenesis is a complex, multifactorial, and multi-stage process of formation of neurons and neuroglial cells from neural stem cells. Neurotrophic factors, such as brain-derived neurotrophic factor (BDNF), nerve growth factor (NGF) and fibroblast growth factor (FGF), play a special role in the regulation of neurogenesis. The fine regulation of neurogenesis, which occurs at all its stages, includes the molecular mechanisms of cellular signaling mediated by S1P and its receptors. In particular, S1P and its receptors affect adult neurogenesis at multiple stages: activation of S1P receptors induces neuroblast proliferation, migration, and morphological changes. Special attention is paid to the interaction of S1P and its receptors with the cellular environment (astrocytes, endothelial cells, and ependyma) of the neurogenic niche and the relationship between S1P metabolism and the expression of neurotrophins, which suggests a key role of S1P in regulating the environment of the neurogenic niche.

## 1. Introduction

Brain plasticity, which enables the adaptation and maintenance of central nervous system (CNS) functions throughout life, is mediated by a series of processes, including neurogenesis, oligodendrogenesis (and the associated remyelination), and angiogenesis. Neurogenesis, as a homeostatic process of new nerve cell formation, occurs most intensively during embryonic and postnatal development [1]. The issue of neurogenesis in the adult human body has long been subject to criticism: it was believed that the concept of continuous neurogenesis in adults lacked sufficient evidence; doubts were raised regarding the localization of neurogenesis and its dynamics in the adult human brain, as well as the methodology and indicators of neurogenesis, etc. However, sufficient data have now been accumulated to show that the human brain’s ability to generate new neuronal and glial cells persists throughout life, and that processes such as memory, learning, adaptive behavior, stress responses, and the formation of psychoemotional states are specifically linked to adult neurogenesis [2,3]. Neurogenesis has been studied in the greatest detail in rodents, as their olfaction is highly developed from an evolutionary perspective, and therefore neurogenesis of olfactory cells occurs actively throughout their entire lives [1].

According to the currently accepted theory of neurogenesis, all cells of the central nervous system—neurons and macroglia—derive from a single type of neural stem cell (NSC) [3,4,5]. To give rise to various types of neurons and glial cells in the central nervous system, neural stem cells undergo specific stages of transformation—proliferation, differentiation (through intermediate forms), migration to specific brain regions, and the integration of mature neurons into the neural network. These processes occur under the complex, multifaceted influence of specific extracellular and intracellular molecular factors, among which neurotrophins and cytokines play a major role, such as brain-derived neurotrophic factor (BDNF), nerve growth factor (NGF), fibroblast growth factor (FGF), neurotrophin-4/5 (NT-4/5), and neurotrophin-3 (NT-3), platelet-derived growth factor B (PDGF-B), interleukin-1 (IL-1), tumor necrosis factor alpha (TNF-α), and others [5,6,7,8]. Pluripotent stem cell regulation by neurotrophic factors forms the basis of modern protocols for generating various types of CNS cells [9].

A critically important process for maintaining homeostasis in the adult brain is remyelination, which is largely driven by oligodendrogenesis—that is, the process by which oligodendrocytes are generated from oligodendrocyte precursor cells (OPCs), which also originate from the neural stem cells (NSCs) [10,11]. Oligodendrocytes play a crucial role in brain physiology and pathology by providing myelination of axons [12,13]. In addition, angiogenesis and the stability of the blood-brain barrier (BBB) are essential for maintaining central nervous system function and facilitating recovery from trauma and ischemic damage [14].

Sphingolipids (SLs)—a class of lipids containing a sphingoid backbone—play a special role in regulating the activity of brain cells, including both neurons and glial cells, under both normal and pathological conditions. Sphingolipids are present in significant quantities in nervous tissue, and in myelin, they account for more than half of all lipids. SLs are essential for nervous system function during the perinatal and early postnatal periods, for its normal function in adults, and for maintaining function during aging [15,16].

One of the key compounds in sphingolipid metabolism is sphingosine-1-phosphate (S1P), which exerts both autocrine and paracrine effects. S1P acts as a signaling molecule within the cell and triggers cascades of events that regulate cell growth, cell migration, inflammation, angiogenesis, and other processes in the brain. Upon being transported into the extracellular space, S1P binds to five specific G-protein-coupled S1PR1–5 receptors, which, in turn, control numerous processes, including synaptogenesis, neurotransmission, neurogenesis, cell proliferation, and migration, ensuring the function of BBB and maintaining brain homeostasis under normal conditions and/or contributing to the development of various pathologies [16,17,18,19,20,21,22]. An understanding of the molecular mechanisms underlying the action of S1P has led to the development of pharmacological agents for the treatment of CNS disorders, the best known of which are fingolimod and siponimod as well as sphingosine kinase (SK1) inhibitors [16,17,21].

Numerous studies have demonstrated the key role of S1P metabolism and its receptors in the signaling of stem cells and precursor cells of various types, including hematopoietic cells [23,24], muscle [24,25], endothelial [24,26], neural [27] and others [19,24,28]. The role of S1P metabolites in embryonic and postnatal neurogenesis has been well studied (see detailed reviews [15,24,29,30]). However, studies examining the role of S1P in the regulation of adult neurogenesis are still relatively scarce. The peak in publications on this topic occurred between 2005 and 2015. Over the past decade, the focus has shifted toward translational research. Nevertheless, recent studies continue to investigate the influence of S1P on the development of neurogenesis in adults [31,32].

The aim of this review is to summarize the evidence pointing to the role of S1P and its receptors in reparative processes in the adult brain. We examine the involvement of S1P signaling in the regulation of neurogenesis, its interaction with neurotrophic factors, and its influence on the cellular components of the neurogenic niche. We also analyze the role of S1P in oligodendrogenesis and angiogenesis, which are integral components of neural tissue repair following injury. A new perspective on the role of S1P in reparative processes in the CNS may open up prospects for the application and development of pharmacological agents that modulate S1P metabolism.

## 2. S1P Metabolism and the Main Signaling Mechanisms

The lipid mediator S1P is an essential component of numerous signaling pathways. Its metabolic precursor, as is the case for most other sphingolipids (sphingomyelin, ceramide-1-phosphate, glycosphingolipids, gangliosides, and many others), is ceramide. It is important to note that ceramide, as an intracellular messenger, possesses proapoptotic properties, and the enzymes involved in its metabolism participate in the transmission of apoptotic signals, whereas S1P, on the contrary, in most cases promotes cell survival, proliferation, and migration. The ratio of ceramide to S1P levels, which is now commonly referred to as the “sphingolipid rheostat,” plays a key role in determining the fate of the cell [15,17,33,34].

**Figure 1:**
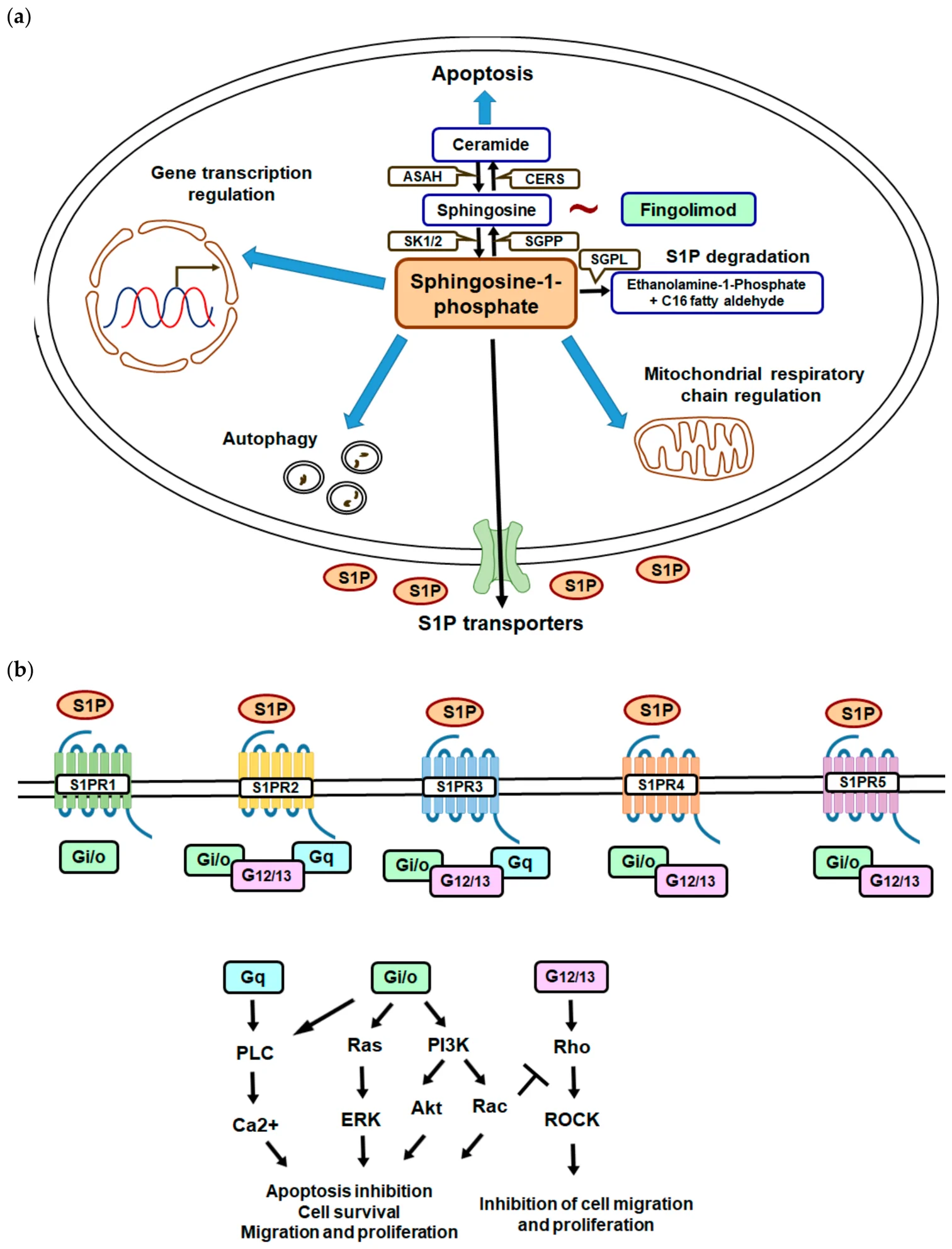
(**a**) Intracellular S1P metabolism and receptor-independent actions. Black arrows indicate S1P metabolism, blue arrows—intracellular/receptor-independent S1P actions; Fingolimod replaces sphingosine, indicated by the red wavy line (S1P–sphingosine-1-phosphate, SGPL–S1P lyase, SK–sphingosine kinase, ASAH–acid ceramidase, CERS–ceramide synthases, SGPP–sphingosine-1-phosphate phosphatase). (**b**) Extracellular S1P signaling mediated by S1PR1–S1PR5 and downstream G protein pathways (S1P–sphingosine-1-phosphate, S1PR1–5–sphingosine-1-phosphate receptors 1–5; Gi/o–alpha subunit of G protein i/o, Gq–alpha subunit of G protein q, G12/13–alpha subunit of G protein family 12/13).

The formation of S1P from ceramide occurs in two stages (Figure 1a). In the first stage, under the action of ceramide-degrading enzymes (alkaline—ceramidases 1-3 (ACER1, ACER2, ACER3), acidic—acid ceramidase (ASAH1), neutral—neutral ceramidase (ASAH2)), ceramide is deacylated to form sphingosine. In the second stage, sphingosine is phosphorylated to form S1P. This reaction is catalyzed by two sphingosine kinases (SK1 and SK2). SK1 is localized primarily in the cytosol, whereas SK2 is found in the mitochondria, endoplasmic reticulum, and nucleus [34]. In response to specific stimuli, SK1 translocates to the plasma membrane, where S1P is synthesized. The reverse process—the dephosphorylation of S1P to sphingosine—is also possible and is catalyzed by S1P phosphatases and nonspecific lipid phosphatases [15,16,20].

The only irreversible reaction is the cleavage of S1P by the enzyme S1P lyase (SGPL), which is localized on the endoplasmic reticulum membrane. S1P is degraded to form phosphoethanolamine and hexadecanal, thereby removing S1P from the sphingomyelin cycle. Thus, the activity and expression of key enzymes—sphingosine kinases, phosphatases, and S1P lyase—are critical points in the regulation of S1P levels and are the focus of research into the molecular mechanisms of S1P action as a lipid messenger [16,17,24].

S1P is involved in various intracellular metabolic pathways and is also transported into the extracellular space (Figure 1a). Established receptor-independent targets include TNF receptor-associated factor 2 (TRAF2), which participates in NF-κB signaling, histone deacetylases HDAC1 and HDAC2, mitochondrial PHB2, and mechanisms of calcium regulation [16,17,18,35]. Nuclear S1P, generated in part by SK2, can directly bind to HDAC1/2 and inhibit their enzymatic activity, thereby increasing histone acetylation and modifying gene transcription [35]. S1P is also capable of inhibiting ceramide formation by acid sphingomyelinase, reducing oxidative stress, and modulating the expression of pro- and anti-apoptotic proteins of the Bcl-2 family and caspase-3. S1P can activate the regulatory proteins p38 and ERK and inhibit JNK in various tissues. S1P, and the S1P-metabolizing enzymes SK1 and S1P-lyase, are also involved in autophagy [16,17].

As a polar molecule, S1P cannot freely diffuse through the lipid bilayer. S1P transport across the cell membrane can occur via several mechanisms: (1) via ABC family transport proteins; (2) through the secretion of SK1 itself into the extracellular space; (3) via the ATP-dependent transport protein spinster homolog 2 (SPNS2); (4) via the protein MFSD2B (major facilitator superfamily domain containing 2B) [17].

S1P is present both in its free form and as part of lipoproteins. In blood plasma, the majority of S1P is bound to apolipoprotein M (apoM) within high-density lipoproteins (HDL) (~50–60%); approximately 30–40% is bound to albumin, and only a small fraction is found in LDL and VLDL [17]. The S1P-apoM complex is a physiologically active form that regulates immune and vascular processes. In cerebrospinal fluid (CSF), S1P is also found in HDL in a complex with apoM [36], and it is in this form—as the S1P-apoM complex—that S1P can cross the BBB from the blood into the brain [31].

A major principle of S1P regulation is its spatial concentration gradient. S1P is maintained at relatively high concentrations in blood through continuous release from erythrocytes and endothelium and from activated platelets, whereas its concentration in tissue interstitial fluids is much lower because of S1P lyase, S1P phosphatases, and lipid phosphatases [37,38]. This gradient is a well-established mechanism guiding immune-cell trafficking and leukocyte–endothelial interactions [37,39,40]. A similar principle may be relevant to local S1P delivery to adult neurogenic niches, although quantitative thresholds for individual stages of adult neurogenesis have not yet been defined.

The response to S1P depends on several interrelated factors: the local concentration of free and carrier-bound S1P, the repertoire and relative expression of S1PR1–S1PR5, which couple to different G proteins, receptor desensitization, internalization and potential G-protein-coupled-receptor (GPCR) oligomerization/heteromerization and signaling bias (biased agonism), whereby distinct ligands or delivery forms stabilize different receptor conformations and preferentially recruit G proteins or β-arrestins [41,42,43]. Structural studies of S1PR1 demonstrate differences between Gi signaling and β-arrestin-biased responses to specific modulators [42], whereas S1PR3 structures reveal determinants of G-protein subtype selectivity [43]. Differences between experimental models may therefore arise not only from the S1P concentration but also from the predominant receptor subtype and the G proteins expressed in the cells studied. Direct evidence that receptor dimerization controls adult neural stem cells is still limited; this mechanism should therefore be considered plausible but not established for adult neurogenesis.

Extracellular S1P can either be degraded by lipid phosphatases (LPPs) on the cell membrane [44] or act as a ligand for five G-protein-coupled receptors (S1PR1–5) [21]. When S1P binds to the receptor, it activates S1PR-coupled G proteins (Figure 1b), which can initiate various signaling pathways depending on the type of G protein α-subunit [19]. The most widespread and well-studied receptor is S1PR1, which is essential for normal development (an *S1PR1* knockout is lethal at the embryonic stage) [45,46].

The cellular response to S1PR activation depends on the receptor type [18]. Activation of S1PR1 and S1PR3 typically leads to the activation of the PI3K/Akt and MAPK/ERK signaling pathways, which promote cytoprotection, cell survival, and proliferation [19,21,24,30]. However, activation of S1PR2 may also activate another signaling pathway—the Rho/ROCK pathway—which leads to the opposite cellular response: inhibition of cell proliferation and differentiation [17,19]. S1PR1 and S1PR3 stimulate the migration of immune cells, whereas S1PR2, on the contrary, inhibits chemotaxis. Activation of S1PR2 in combination with the Nogo-A reticulon suppresses neurite growth and synaptic plasticity [17,47]. Thus, a cell’s sensitivity to circulating S1P and the nature of its response are determined by the pattern of receptor expression [24,48]. The predominance of S1PR1 in tissues likely explains the frequently observed proliferative and anti-apoptotic effects of S1P, whereas other receptors perform more specialized functions. For example, S1PR5 expression is characteristic primarily of CNS cells, where this receptor plays a special role in the development and function of oligodendrocytes and neurons [18,19].

S1P receptors are widely expressed in the nervous system, which has a high sphingolipid content; they exhibit cell specificity and their expression patterns vary depending on the cell type, developmental stage, or functional state (for a special review of S1P receptors in the CNS, see [19,21]). CNS cells primarily express S1PR1, S1PR2, and S1PR3; numerous studies have shown that S1PR1 is the most prevalent receptor in brain cells, as well as throughout the entire body [27,49,50]. S1PR4, along with S1PR1–3, is expressed by endothelial cells, including endothelial cells of cerebral capillaries, and plays a key role in regulating the BBB [48]. It is worth noting that S1PR5 is specific to CNS cells and is of paramount importance for the development and function of both neurons and oligodendrocytes, as will be discussed in more detail later [45,49].

Neurons typically express the S1PR1, S1PR2, S1PR3, and S1PR5 receptors, with S1PR1 and S1PR5 being more common in precursor cells [24,27,50], while S1PR1 and S1PR3 predominate in mature neurons. The action of S1P via these receptors stimulates neurite outgrowth and neurotransmitter secretion, reduces excitotoxicity, and can increase glutamate release in hippocampal neurons [17]. S1P receptors play an important role in regulating the stability and integrity of BBB, and their dysfunction is associated with increased barrier permeability in various pathological conditions [19,48,51].

Based on an understanding of the physiological role of S1P, a number of pharmacological agents have been developed. The best-known are S1P receptor agonists—sphingosine analogs—such as fingolimod (FTY720), siponimod (BAF312), ponesimod, and others [49,52,53,54]. Once in the body, fingolimod is phosphorylated by SK2; the resulting active metabolite, fingolimod phosphate—a structural analog of S1P—is capable of binding to S1P receptors (with the exception of S1PR2) [52,53,54]. These medications are used to treat multiple sclerosis [54], and are being tested for the treatment of Alzheimer’s disease [53], mental [55], tumor-related [34,56] and inflammatory diseases [54], and are also widely used in scientific research to model S1P metabolic processes [52,54]. Research is also underway to identify and synthesize new pharmaceuticals based on the action of S1P [57,58].

## 3. Role of S1P and Its Receptors in Neurogenesis

Neurogenesis is currently defined as the complex process by which new nerve cells are formed from multipotent precursor cells—neural stem cells. The following main stages of neurogenesis are distinguished: NSC activation, proliferation, lineage commitment, survival and selection of progeny, neuroblast migration, maturation, and final functional integration into the neuronal network. Detailed reviews on adult neurogenesis can be found in the following works [3,4,5].

Neurogenesis is most active during the embryonic period (when there is active proliferation of neural stem cells and precursor cells, and the nervous system is forming), declining sharply after birth. In adult organisms, this process proceeds at a very slow rate and is limited to specific regions: the subventricular zone (SVZ) in the brain ventricles and the subgranular zone (SGZ) of the hippocampus; in addition, neurogenic processes have been detected in the hypothalamus, the nuclei of the nigrostriatal system, the amygdala, and certain regions of the cortex [3,5].

Numerous in vivo and in vitro studies have confirmed the key role of the SK/S1P/S1PR metabolic axis in neurogenesis during the earliest stages of mammalian embryogenesis [16,27,59,60]. Genetic rodent models have shown that abnormalities in S1P metabolism are often lethal to embryos. In mice with a double knockout of the *SK1* and *SK2* genes, increased apoptosis and reduced mitosis were observed in neuroepithelial cells, leading to defects in neurulation (failure of neural tube closure) and angiogenesis, resulting in embryonic death [59]. Interestingly, mice with a knockout of *SK1* alone were viable but exhibited reduced SK activity and low blood S1P concentrations [61], which, according to the authors, is due to compensatory SK2 activity. S1PR1 expression is essential in the early stages of development: a knockout of this gene leads to embryonic death due to impaired vasculogenesis [46].

The influence of sphingosine-1-phosphate lyase deficiency has been the most extensively studied: hereditary mutations in the *SGPL1* gene encoding this enzyme were classified in 2017 as a distinct inherited disorder, sphingosine-1-phosphate lyase insufficiency syndrome (SPLIS), NPHS14 [62]. S1P lyase catalyzes the sole irreversible degradation reaction of S1P, removing it from the sphingomyelin cycle. Its deficiency leads to the accumulation of S1P, sphingosine, and ceramide, which causes disturbances in autophagy, protease activity, calcium homeostasis, and the clearance of aggregation-prone proteins. SPLIS is classified as an atypical sphingolipidosis; over 30 mutations associated with the *SGPL1* gene have been identified [63].

For the normal development of the embryo’s central nervous system, the homeostatic functioning of both the enzymes involved in S1P metabolism and its receptors is essential [16,24]: starting from the early stages of rat embryo development, the neuroepithelium and various brain regions express S1PR1–3 and S1PR5 receptors [50,64]. Furthermore, during embryonic neurogenesis, intense expression of S1PR1 is observed in the ventricular zone of the neocortex, as well as in the hippocampus, olfactory bulb, ganglion cell layer, and other regions. S1PR1 expression is particularly high in the proliferative zones (cortex and other regions) of the embryonic brain, while S1PR2 and S1PR3 expression is significantly lower [27]. Inhibition of SK1 and S1PR1, in turn, blocks neurogenesis [65].

### 3.1. The Potential Role of S1P and Its Receptors in Neural Stem Cells and Neuroprogenitors in Adults

According to the current concept, the adult NSC pool is derived from slowly proliferating embryonic precursors, maintaining a similar transcriptional profile [2]. NSCs in the adult body can remain in a state of prolonged proliferative quiescence, without entering the differentiation stage, and form so-called quiescent NSCs (qNSCs), which allows the pool to be maintained throughout life [66]. Upon activation, qNSCs enter the cell cycle and become active NSCs (aNSCs) [4]. Both quiescent and activated NSCs coexist in the neurogenic niche at any given time. Adult NSCs are capable of generating intermediate subtypes of progenitor cells (neuroblasts), from which neurons and glial cells (astrocytes, ependymal cells) may subsequently develop. The resulting intermediate progenitors (neuroblasts) undergo stages of proliferation, selection (apoptosis of some cells), differentiation, migration, maturation, and, finally, integration into neural networks [66].

Different stages of neuronal cell development are characterized by the expression of specific markers. For example, Nestin and Sox2 are widely used as markers of neural stem cells in both the embryonic and adult brain [49]. The co-expression of CD133 (Prominin), a stem cell marker, and GFAP (glial fibrillary acidic protein), a specific astrocyte protein, is a characteristic marker of niche astrocytes that function as neural stem cells in the subventricular zone [67].

Various signaling pathways are involved in maintaining the homeostatic processes of neurogenesis, the key one being sphingolipid metabolism and, in particular, S1P. The question of the similarity between the molecular mechanisms of embryonic and adult neurogenesis remains open. The role of S1P in embryonic and early postnatal neurogenesis has been studied much more extensively than in the adult brain. Therefore, findings from developmental models are used here only to identify mechanisms that may also operate in adults. In different cell and animal models, S1P acts mainly through its receptors, especially S1PR1, and activates ERK and PI3K/Akt signaling. It can be hypothesized that S1P metabolism is a key regulatory mechanism of neurogenesis in both the embryonic and adult brain, initiating similar signaling cascades in both cases.

The involvement of S1P and its receptors in the regulation of neurogenesis has been demonstrated in cultures and models of rodent and human neural progenitor cells [24,31,52,64].

Quantitative analysis showed that in the neurogenic niche of the SVZ in adult mice, S1PR1 is expressed on NSCs to a significantly greater extent than on astrocytes and endothelial cells [31]. Interestingly, S1P had no proliferative effect on quiescent NSCs: for example, Codega et al. showed that S1P did not stimulate, but rather reduced, the division of quiescent NSCs (qNSCs), without affecting the number of already activated NSCs (aNSCs) [67]. The authors did not investigate the receptor profile of qNSCs; it is possible that the absence of receptors or, conversely, excessive intracellular accumulation of S1P could have shifted the “sphingolipid rheostat” toward pro-apoptotic ceramide.

In subsequent stages, high expression of S1P receptors and a significant influence of S1P on neuronal cell function are observed [24]. S1P induces proliferation and transient cell-cell aggregation in neural progenitor cells from embryonic rat hippocampus via ERK and Rho/ROCK signaling [64]. In olfactory ensheathing cells, S1P promotes proliferation through S1PR1 and RhoA/YAP signaling [32].

The role of S1P has also been confirmed in human neural progenitor cells. Neural progenitor cells of epithelial origin in the human embryo express S1P receptors [19,30,50]. In cultured human neural progenitor cells, S1P increased cell survival, while its effect on neuronal differentiation was inhibitory [30]. The PI3K/Akt signaling pathway is involved in the regulation of proliferation and differentiation of human neural progenitor cells [68,69]. In a study by Guo et al., it was shown that the pharmacological agent 3K3A-APC stimulates the proliferation, migration, and differentiation of neural stem cells specifically through S1PR1 and the subsequent activation of PI3K/Akt [65].

S1PR1 is also essential for neuroblast migration: S1P induces the migration of neural stem cells/progenitors, a process in which receptors 1 and 3 are involved—specific inhibition of these receptors leads to the cessation of migration [70,71]. S1PR1 helps maintain intercellular contacts between neuroblasts during their chain migration along the rostral migratory tract [72].

At later stages, changes in receptor expression may become increasingly important. Many neural progenitors show relatively high S1PR1 expression, whereas maturation is accompanied by a greater relative contribution of S1PR2 and S1PR3 [17,71]. Through these receptors, S1P can influence neurite growth or retraction, synaptic plasticity and neurotransmission. Therefore, the same extracellular S1P concentration may produce different responses at different developmental stages because the receptor repertoire and coupled G proteins change during maturation.

In adult stroke models, pharmacological modulation of S1P receptors has been associated with increased neurogenesis and functional recovery [28]. In another study, circulating apoM-bound S1P reached the adult SVZ and altered the niche [31]. These findings constitute direct evidence that S1P signaling can affect the adult neurogenic system. However, causal evidence is still much more limited for some late stages (particularly long-term functional integration of newly generated neurons) than for proliferation and migration.

### 3.2. The Potential Role of S1P and Its Receptors in the Neurogenic Niche

Adult neurogenesis occurs in specialized microenvironments—neurogenic niches—which constitute a complicated cellular and extracellular complex [73]. The cellular component of the niche includes quiescent and active NSCs, neuroprogenitors at various stages of development, as well as various types of supporting cells: astrocytes, microglia, endothelial cells, and ependymal cells [2,4]. These cells produce growth factors, extracellular matrix components, and signaling molecules, creating a unique microhomeostasis. Interaction with the circulatory system and cerebrospinal fluid via the endothelium and ependyma allows circulating biomolecules to access the niche, which can modulate the intensity of neurogenesis [31,74].

#### 3.2.1. S1P and the Cellular Environment of the Neurogenic Niche

The SK/S1P/S1PR metabolic axis may influence neurogenesis not only directly, by acting on progenitor cells, but also indirectly—through the regulation of other niche cells’ functions and the synthesis of neurotrophins.

***Astrocytes*** are key regulators of all stages of neurogenesis [66,73]. They exert a significant influence on neurogenesis through the release of neurotrophins and cytokines, which can directly affect neurogenesis as well as a wide variety of other CNS processes, including neurotransmission, neurotoxicity, and the induction of apoptosis, which can directly affect behavior and brain damage/pathology of various etiologies [66,75].

Astrocytes primarily express S1PR1, S1PR2, and S1PR3, and their expression changes in response to inflammatory conditions (e.g., neuroinflammation) [19]. Astrocyte activation is directly linked to S1P signaling: human and mouse astrocytes express significantly more S1P receptors than neurons [76]. Furthermore, the S1PR1 and S1PR2 receptors are essential for the induction of trophic factor genes in astrocytes [76], which indicates their key role in the production of these factors. It is likely that a significant portion of S1P’s influence on the neurogenic niche is mediated through the modulation of astrocyte function.

***Microglia*** are resident cells of the immune system and play an active role in neurogenesis. Activated microglia support and modulate neurogenesis through the secretion of cytokines and NTFs, the induction of apoptosis, and the phagocytosis of apoptotic cells, thereby promoting the proliferation, differentiation, and survival of neuroprogenitors [5,66,73]. Activated microglia exist in two polarized states: the M1 phenotype, which is a pro-inflammatory (neurotoxic) state associated with the release of pro-inflammatory chemokines and cytokines [66]; the M2 phenotype is anti-inflammatory; these cells produce anti-inflammatory mediators (resolvins, protectins, and maresins) [5].

Microglia cells express S1PR1–3 depending on the stage of activation, the age of the organism, and other factors, with S1PR1 being significantly predominant [77]. S1P receptors play a significant role in microglial signaling and function. Activation of S1P receptors by fingolimod and other receptor agonists reduces microglial activation [7,19,55].

There is a possible connection between S1P signaling, neurogenesis and neuroinflammation. S1P controls lymphocyte trafficking, endothelial permeability and inflammatory mediator production, and SK1/S1P signaling in activated microglia can enhance expression of pro-inflammatory cytokines [78]. Conversely, functional S1PR1 antagonists such as fingolimod reduce lymphocyte entry into the CNS and, in several models, attenuate microglial and astrocytic reactivity [7,19,54,58]. Neuroinflammation itself has bidirectional effects on adult neurogenesis: a transient, controlled inflammatory response may facilitate clearance of damaged cells and reparative signaling, whereas chronic inflammation and sustained elevation of IL-1β, TNF-α, and related mediators generally suppress proliferation, neuronal differentiation, and the survival of newborn neurons [5,79]. Thus, S1P-mediated regulation of neurogenesis through inflammation may change direction over time and depends on the dominant cellular and receptor components of the S1P system.

***Endothelial cells*** are the primary structural component of the BBB and an important component of the neurogenic niche [73]. Brain endothelial cells express S1PR1, S1PR2, and S1PR3, which are essential for maintaining the integrity of the BBB and regulating its permeability [19]. In addition, it has been found that endothelial cells, including those in the brain capillaries, express S1PR4, which also plays a role in maintaining the function of the BBB [48]. Activation of the SK1/S1P axis is involved in the regulation (pyroptosis) of BBB endothelial cells via the ERK1/2 signaling pathway [22]. In certain pathological conditions (such as meningitis), disruption of the BBB also involves S1PR1 [80].

The endothelium, along with the ependyma, plays a crucial role in transporting S1P from the blood into neurogenic niches. A study by Choi et al. demonstrated that when an isotope-labeled apoM-S1P complex is administered intravenously to mice, S1P crosses the BBB, accumulates in the SVZ, and stimulates neurogenesis. Furthermore, a decrease in the blood concentration of the apoM-S1P complex correlates with a reduction in S1P concentration in the SVZ [31]. In addition, under the influence of S1P, endothelial cells can induce the expression of the trophic factor VEGF-C via a signaling pathway associated with FGF-1/FGFR-1 [81].

***The ependyma***, a layer of glial cells lining the ventricles of the brain, facilitates the circulation of cerebrospinal fluid through its numerous cilia [74]. Ependymocytes are also equipped with microvilli that absorb CSF. The ependyma is an important structural and trophic component of the neurogenic niche, regulating the flow of substances from the CSF into the neurogenic niche [74,82]. In pathological conditions (for example, in the experimental autoimmune encephalomyelitis model, tEAE), ependymal cells undergo significant morphological changes [83]. Significant changes in ventricular ciliated cells occur at an early stage in response to tEAE, suggesting that these cells play a role in signaling from SVZ stem cells not only during development and/or aging but also during inflammatory demyelination [83].

On the one hand, S1P present in the CSF enters the brain—including neurogenic niches—via ependymal cells. On the other hand, the functionality of ependymocytes and their self-renewal are regulated by S1P metabolites. In a model of Alzheimer’s disease (*Apom*−/−mice), abnormalities in the structure of ependymal cilia and the resulting disruptions in CSF circulation have been demonstrated, which are corrected by activation of the apoM-S1P-S1PR signaling pathway [31]. Given the common origin of ependymal cells and NSCs [74,82], it can be hypothesized that S1P is also involved in regulating ependymal development.

#### 3.2.2. S1P and Neurotrophic Factors in the Regulation of the Neurogenic Niche

Neurotrophins (NTFs) are a heterogeneous group of polypeptides (BDNF, NGF, GDNF, FGF, PDGF-B, VEGF, etc.) that perform a wide range of functions in the nervous system: regulating the processes of growth, differentiation, and maintenance of neural tissue viability [8], as well as the regulation of synaptogenesis, which collectively contributes to the survival of neurons and neural stem cells, synaptic plasticity, and memory formation—factors that, taken together, ultimately account for the significant role of NTFs in neuroplasticity processes. The main (though not the only) producers of NTFs in the brain are astrocyte and microglial cells [66].

Neurotrophins are key extracellular regulators of neurogenesis [6,73,79,84]. The most extensively studied is BDNF, which is involved in all stages of neurogenesis—from progenitor cell differentiation to the integration and survival of mature neurons [3,84]. Neurotrophins transmit signals via two types of receptors: specific tyrosine kinase receptors (TrkA for NGF, TrkB for BDNF and NT-4, TrkC for NT-3) and the low-affinity p75NTR receptor, which belongs to the TNF receptor superfamily. The p75NTR can activate both proliferative and apoptotic pathways, as well as modulate Trk receptor signaling [3,6,84].

There is a close bidirectional relationship between S1P metabolism and neurotrophins.

***S1P initiates the expression of neurotrophic factors***. Over the past few decades, many researchers have demonstrated the direct influence of S1P on the expression and activity of neurotrophic factors [18]. This connection has been studied most extensively in astrocytes—the primary producers of neurotrophins in the brain [18,76,85,86]. Tran et al. demonstrated that S1P induces the expression of genes and the synthesis of proteins for the growth factors BDNF, heparin-binding EGF-like growth factor (HB-EGF), leukemia inhibitory factor (LIF), and platelet-derived growth factor B (PDGF-B) in cultures of primary mouse and human astrocytes, as well as in U251 astrocytoma cell lines [76]. Activation of S1P receptors in a human astrocytoma cell line induces the expression of mRNAs for the neurotrophic factors LIF, IL-11, and HB-EGF, followed by the secretion of these proteins [85], whereas inactivation of the S1PR1 and S1PR2 receptors inhibits the induction of these trophic factor genes [76].

S1P also stimulates the synthesis of BDNF, HB-EGF, LIF, and PDGF-B in primary neuronal cultures and hippocampal slices [76]. Activation of S1P receptors increases BDNF expression in cortical neurons of mice [87], and exogenous S1P induces the synthesis of the neurotrophic factor LIF, which promotes the survival of hippocampal neurons [76]. The addition of S1P to neuroblastoma culture medium has an effect similar to that of GDNF—it stimulates GAP43 transcription (GDNF-induced neuronal phenotype) [88].

The ability of astrocytes to synthesize NTFs more actively in response to S1P correlates with higher expression of S1P receptors on their surface compared to neurons [76]. S1PR1 and S1PR2 are primarily involved in the induction of NTF expression (BDNF, HB-EGF, LIF); in some cases, such as for PDGF-B synthesis, activation of all three receptors—S1PR1, S1PR2, and S1PR3—is required [76]. In addition, other cells of the neural niche are also capable of synthesizing neurotrophins: as previously noted, S1P stimulates the expression of the trophic factor VEGF-C by endothelial cells [81].

Modulation of S1P receptors by fingolimod increases BDNF levels not only in vivo but also in vitro: increased BDNF mRNA expression and protein levels were observed when fingolimod was added to the culture medium in cultured neurons, and when fingolimod was injected into the hippocampus, cerebral cortex, and striatum of control wild-type mice and mice modeling Rett syndrome. In both cultured neurons and the brains of model animals, this was accompanied by increased ERK1/2 phosphorylation [89]. Administration of S1P to animals (a mouse corneal epithelial injury model), as well as treatment of mouse trigeminal ganglion neurons with S1P, induces the expression of NGF, BDNF, and GDNF [90]. Blocking S1PR2 with the highly selective inhibitor JTE-013 led to increased BDNF synthesis in the hippocampus of rats in a model of hepatic encephalopathy [7].

Thus, S1P itself, the activation of its receptors, the S1P receptor agonist fingolimod, and a specific, targeted S1PR2 inhibitor all increase the expression of NTFs by astrocytes. One possible mechanism for regulating NTF gene expression is the modification of histone proteins: S1P specifically bound to the histone deacetylases HDAC1 and HDAC2 and inhibited their enzymatic activity [35]. In turn, HDAC inhibition increases expression of neurotrophic factor BDNF [91]. Thus, S1P can indirectly influence *BDNF* expression through both intracellular S1P and the S1PR1.

***Cross-regulation between the S1P signaling pathway and neurotrophins:*** It has been shown that S1P is involved in neurotrophin signaling. This is because, in addition to acting as a trigger for neurotrophin gene expression, S1P functions as a signaling molecule that transmits signals from various trophic factors and cytokines. In the case of tyrosine kinases, S1P acts as a signaling messenger during the activation of TrkA (NGF) [92] and TrkB (BDNF) [7] receptors. The role of ceramide, a precursor of S1P, in p75NTR receptor signaling has long been known [93], which led Nicol to hypothesize that S1P itself is also involved in this cascade [94]. It is possible that S1P, in turn, regulates the activity of TRAF2 [95], which acts as a regulator of the p75NTR receptor. Thus, S1P may function as a secondary messenger upon the binding of a neurotrophin (e.g., BDNF) to the p75NTR receptor.

The findings support the hypothesis that S1P is an intracellular secondary messenger in the NGF-induced increase in neuronal excitability [96].

The molecular mechanisms underlying S1P-mediated signaling that lead to the expression of NTF genes include the phosphorylation of ERK1/2 and the subsequent induction of the transcription factors EGR1 and c-FOS, which regulate the promoters of the BDNF, HB-EGF, LIF, and PDGF-B genes [76,87]. Inhibition of S1P receptors leads to a reduction in FOS induction and also decreases JUN phosphorylation in glioblastoma and astrocyte cultures [76].

It has been established that NTFs, in turn, regulate S1P metabolism. Many growth factors (EGF, PDGF, IGF, VEGF, NGF, TGF) act as activators of SK1 and stimulate S1PR synthesis in various cell types [24]. For example, FGF is capable of regulating the extracellular transport of S1P from astrocytes [24], which may influence the state of the neurogenic niche. In neuroblastoma cells, GDNF increases *SK1* transcription, leading to increased S1P secretion, and phosphorylates the S1PR1 and S1PR3 receptors [88].

NGF promotes the translocation of SK1 to the plasma membrane and activates the S1PR1 and S1PR2 receptors [97]. The expression of the trophic factor VEGF-C, produced by endothelial cells in response to S1P, occurs via a signaling pathway associated with FGF-1/FGFR-1 [81].

Thus, there is a complex network of mutual regulation between the S1P signaling system and neurotrophins, which influence the regulation of nerve cell and glial function. It is therefore reasonable to assume that the interaction between S1P and NTF metabolism may be directly involved in neurogenesis, which has indeed been demonstrated in a number of studies on neuroprogenitors.

For example, a study of the cross-talk between S1P and LIF (leukemia inhibitory factor) showed that LIF influenced S1P’s ability to regulate the ERK and Akt signaling pathways in human astrocytes and neural progenitor cells [30,76]. In a study by Harada et al., it was shown that in cultured neural progenitor cells, S1P enhances the activity of telomerase in a manner similar to that of FGF-2, one of the key neurotrophins regulating neurogenesis [64].

A synergistic effect of S1P and LIF/bFGF on the survival and proliferation (via activation of the ERK/MAPK and Akt signaling pathways) of human neuroprogenitors has been demonstrated [30]. Furthermore, in cortical astrocytes of rat embryos, S1P dose-dependently stimulates the expression of GDNF mRNA and the synthesis of the GDNF protein itself [86]. Some NTFs (e.g., TGF-β) activate the same signaling pathways (Rho/p160ROCK1) as S1P, which are responsible for morphological changes and cell aggregation [50,98]. S1P promotes the production of GDNF and cytokines by triggering the SK1/S1P signaling pathway, e.g., TNF [99], as well as the steroid hormone estrogen [24].

**Figure 2:**
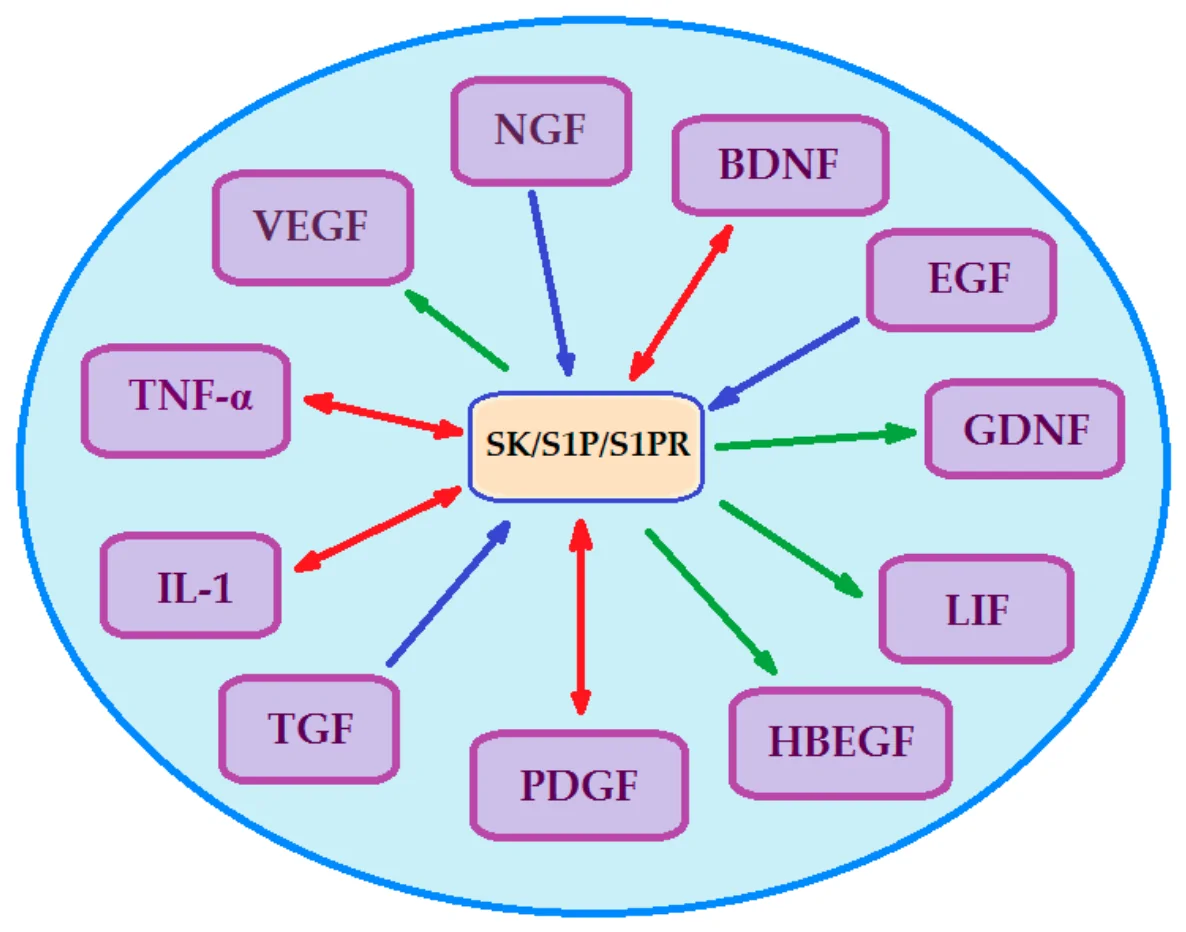
The bidirectional relationship between S1P metabolism and the neurotrophic factors: activation of the metabolic axis S1P/S1PR initiates the expression of neurotrophic factors (green arrows); neurotrophic factors act as activators of SK1 and stimulate S1PR synthesis (blue arrows); mutual regulation between the S1P signaling system and neurotrophins (red arrows); (BDNF–brain-derived neurotrophic factor, GDNF–glial cell line-derived neurotrophic factor, EGF–epidermal growth factor, IL-1–interleukin-1, HB-EGF–heparin-binding EGF-like growth factor, LIF–leukemia inhibitory factor, NGF–nerve growth factor, PDGF-B–platelet-derived growth factor B, TGF–transforming growth factor, TNF-α–tumor necrosis factor alpha, VEGF–vascular endothelial growth factor).

It is clear that in cells of the nervous system, including neural progenitor cells, S1P plays a significant role in regulating NTFs, and conversely, neurotrophins have a substantial effect on S1P metabolism; therefore, through the involvement of S1P receptors and their interaction with neurotrophins (Figure 2), regulation of the neurogenic niche may also occur.

**Figure 3:**
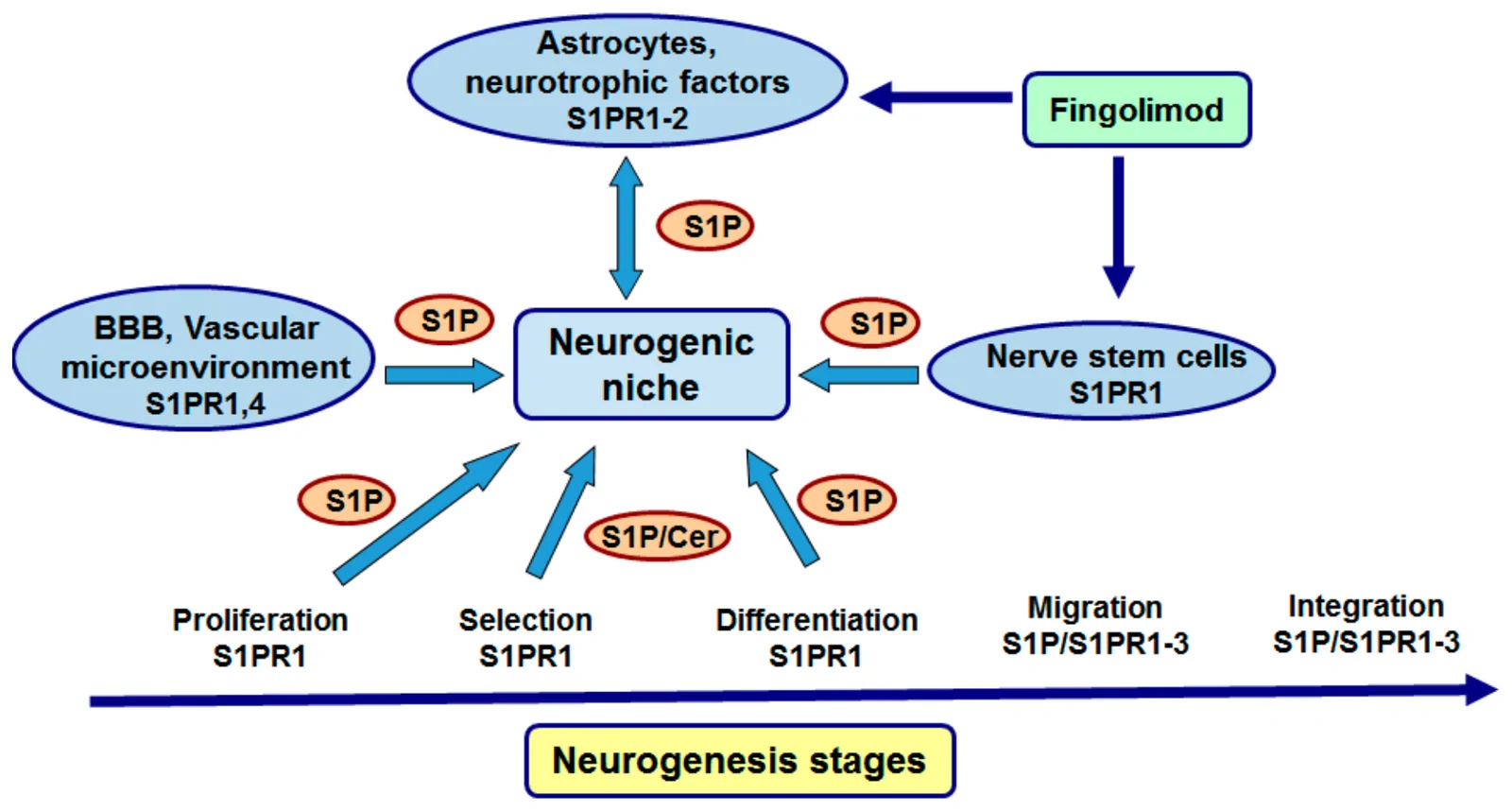
The potential role of S1P and its receptors at different stages of neurogenesis (the arrows are light blue). The action of fingolimod is indicated by dark blue arrows (BBB–blood-brain barrier, Cer–ceramide, S1P–sphingosine-1-phosphate, S1PR1–5–sphingosine-1-phosphate receptors 1–5).

Thus, S1P plays a multifaceted role in the neurogenic niche, acting both as a direct regulator of NSC and progenitor activity and as a key modulator of the microenvironment through its control of astrocyte, endothelial, and ependymal functions, as well as neurotrophin production (Figure 3).

## 4. The Role of S1P and Its Receptors in Oligodendrogenesis

Oligodendrocytes (OLDs), which form the myelin sheath around axons, are critically important for the functioning of the central nervous system [12,13]. They originate from oligodendrocyte precursor cells (OPCs), which, in turn, are derived from NSCs during the late prenatal and early postnatal periods [10,11,13,100]. In the adult brain, there is a large pool of OPCs distributed throughout the parenchyma (5–10% of all CNS cells) [100]. These cells express the NG2 marker (a membrane chondroitin sulfate proteoglycan), which is later replaced by O4+ [11]. OLD are essential for maintaining normal myelin plasticity and ensuring remyelination in pathological conditions (multiple sclerosis, trauma, ischemia). During pathological processes, OLD are activated, proliferate, migrate to the site of injury, and differentiate into mature myelinating oligodendrocytes.

**Figure 4:**
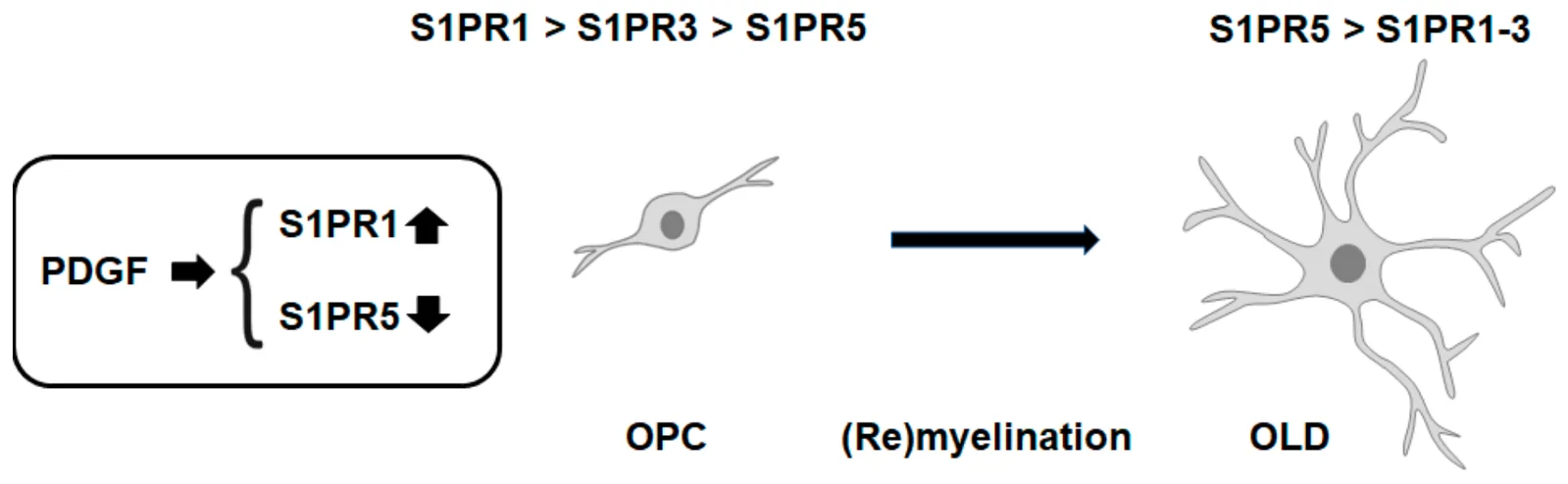
Changes in S1P receptor types depending on the stage of oligodendrocyte development: OPCs predominantly express S1PR1 and, to a lesser extent, S1PR5 and S1PR3; mature oligodendrocytes, in contrast, express mainly S1PR5 and significantly less S1PR1–3; PDGF increases S1PR1 expression and decreases S1PR5 expression.

It has been shown that the types of S1P receptors change depending on the stage of oligodendrocyte development (Figure 4): OPCs predominantly express S1PR1 and, to a lesser extent, S1PR5 and S1PR3; mature oligodendrocytes, in contrast, express mainly S1PR5 and significantly less S1PR1–3 [17,18,19,45,49]. This change in the receptor profile also determines a change in the cellular response. In OPCs, S1PR activation (e.g., by low doses of fingolimod) stimulates survival, proliferation, and migration [18,45]. At the same time, high concentrations of S1P may exert a proapoptotic effect on mature oligodendrocytes, mediated by the accumulation of sphingosine and ceramide [18].

S1P regulates the development of OPCs, their morphological maturity, and the early stages of myelination [18]. Deletion of the *S1PR1* gene in mice leads to a delay in the differentiation of OPCs into mature oligodendrocytes and a decrease in the level of myelin basic protein (MBP). Conversely, stimulation of S1PR1 with the agonist SEW2871 reduced the phosphorylation of Akt, ERK, and PAK1/2 in OPCs [101].

The role of S1PR1 in remyelination has been confirmed in a cuprizone-induced demyelination model [18,49]. In the CNS, there is also mutual regulation of S1P and growth factor signaling; for example, PDGF increases S1PR1 expression and decreases S1PR5 expression [102]. The involvement of SK1 in the neurotrophin-3 (NT-3) signaling pathway was demonstrated in cultured oligodendrocyte progenitors [24].

The regulation of S1P receptor expression in OPCs may also be mediated by astrocytes, which act as cellular mediators of myelination: they regulate S1PR3 expression in OPCs and promote their proliferation [17,18]. Thus, S1P receptors play a decisive role at all stages of oligodendrocyte life, and the shift in their expression from S1PR1 to S1PR5 during maturation is a key mechanism that switches cellular responses.

## 5. The Role of S1P and Its Receptors in CNS Angiogenesis

In addition to contributing to the maintenance of the glial–endothelial barrier and the functionality of the neurogenic niche’s endothelium, S1P plays an important role in angiogenesis—the process of forming new blood vessels, which is essential for tissue repair following ischemia or trauma. During embryogenesis, the SK/S1P/S1PR axis is critical for the formation of the vascular system. Knockout of *SK1*/*SK2* causes abnormalities in angiogenesis [59], while the absence of S1PR1 leads to abnormal endothelial development and embryonic death [61]. Overall, S1PR1–3 receptors are essential for the normal development and function of the circulatory system [20,46], including the development of angiogenesis in the embryonic brain [27]. In adults, vascular endothelial cells express S1PR1–3 receptors, which promote vascular growth and influence vascular permeability [19]. S1P promotes differentiation, cell proliferation, endothelial cell survival, and capillary tube formation [103].

Angiogenesis in the adult body is driven, in part, by circulating endothelial progenitor cells (EPCs) derived from bone marrow stem cells [14]. The key role of the SK1/S1P/S1PR signaling pathway in the proliferation and migration of EPCs and their formation of capillaries has been demonstrated [104].

Under hypoxic conditions, S1P enhances the sprouting and directed migration of EPCs, and the combined action of S1P and VEGF potentiates their proliferation and reparative properties [105]. In addition, S1P interacts with the growth factors FGF-2 and VEGF [26], and itself induces the expression of VEGF-C in endothelial cells [26,81].

Thus, it has been shown that S1P is a proangiogenic factor that promotes the formation of new blood vessels in the brain, maintains endothelial integrity, and ensures the stability of the BBB.

Therefore, the body of evidence suggests that the SK/S1P/S1PR regulatory axis is directly involved not only in embryonic but also in adult neurogenesis, as well as in oligodendrogenesis and angiogenesis (Table 1).

## 6. Discussion

This review highlights SK/S1P/S1PR metabolism as a potential key regulatory signaling axis in adult neurogenesis and the reparative processes of the adult brain as a whole. Particular attention is paid to the role of S1P/S1PR signaling in the neurogenic niche as an integrated cellular system.

Although the extent to which the molecular mechanisms of embryonic and adult neurogenesis overlap remains a matter of debate, the commonality of the signaling pathways involved (in particular, the activation of ERK, PI3K/Akt under the influence of S1P) allows us to suggest that S1P metabolism is one of the conserved regulatory mechanisms of neurogenesis at different stages of ontogenesis.

The SK/S1P/S1PR signaling axis has been shown to be involved in all stages of neurogenesis—from NSC proliferation to the integration of mature neurons. This finely tuned regulation occurs both directly, through the activation of intracellular signaling pathways within the progenitor cells themselves, and indirectly through the modulation of the neurogenic niche microenvironment. S1P influences the functions of astrocytes, endothelial cells, and ependymal cells, and also interacts closely with the neurotrophic factor system, stimulating their expression and participating in signal transduction. This multifaceted role of S1P in the niche allows it to be considered one of the key regulators of the neurogenic microenvironment.

Available data suggest that the influence of the SK/S1P/S1PR axis cannot be described as uniformly proneurogenic. This influence depends on the cell type, receptor expression, and the stage of neurogenesis. In NSCs and neural progenitors, the most frequently implicated downstream pathways are ERK, PI3K/Akt, and Rho/ROCK. S1P signaling may also affect neurogenesis indirectly by changing astrocyte, endothelial, and microglial functions and by regulating neurotrophic and inflammatory gene expression. These pathways recur in studies of several cell types. Nevertheless, activation of the same pathway does not necessarily produce the same biological response, because S1PR expression and the maturation state of the responding cell differ between experimental systems.

In the course of the transition from NSC quiescence to activation, S1P may contribute either to the maintenance of qNSCs or to their activation [31,67]. During proliferation and early differentiation, S1PR1/PI3K/Akt/ERK-dependent pro-survival effects are more commonly observed [30,50,65]. As cells undergo selection, receptor signaling is complemented by the balance between ceramide and S1P, which can shift the probability of survival versus apoptosis. The most direct migration data implicate S1PR1 [70,72]. At the maturation and integration stages, a role for S1P is plausible based on neurite and synaptic plasticity studies but is less firmly established. Apparently, S1P is characterized by stage-specific and sometimes bidirectional regulation.

The concept of the sphingolipid rheostat should be taken into account. Accumulation of particular ceramide species can increase cellular stress and apoptosis and has been associated with neurodegenerative states, whereas a relative predominance of S1P is often accompanied by cell survival and proliferation [106,107]. In tumors, however, increased S1P signaling can promote pathological proliferation and invasion [34]. Thus, the reparative effect cannot be explained by an increase in S1P alone. It depends on compartment- and cell-specific balance among S1P, sphingosine, and ceramide species.

Another unresolved question remains the concentration and localization of S1P. The blood-to-tissue gradient is well established in the immune system [37,39], and delivery of apoM-bound S1P to the SVZ shows that the circulating S1P pool can influence the adult neurogenic niche [31]. However, local S1P concentrations immediately surrounding individual NSCs, their changes after injury, and the concentration ranges at which signaling through S1PR1, S1PR2, and S1PR3 predominates are unknown. The possible contribution of receptor internalization, carrier-dependent signaling, receptor heteromerization, and biased agonism must also be established in native neural tissue since most evidence for these mechanisms was obtained in recombinant systems [41,42,43].

## 7. Conclusions

The long-held belief that the brain is incapable of regenerating has now been disproved. Research over the past decades has convincingly demonstrated the existence of processes of continuous cell renewal—neurogenesis, oligodendrogenesis, and angiogenesis—in the central nervous system of adult mammals, which collectively ensure neuroplasticity and underlie reparative processes. Adult neurogenesis is a complex, multi-stage process, the dysfunction of which is associated with a wide range of CNS pathologies [4,108].

Nevertheless, it is clear that the SK/S1P/S1PR metabolic axis plays a crucial role in the reparative processes of the adult brain. An in-depth study of the molecular mechanisms underlying its action, both from a fundamental perspective and in the context of practical application, is one of the pressing challenges in modern neurobiology and neuropharmacology.

## Figures and Tables

**Table 1:** The effect of S1P and its receptors on the different types of brain cells.

Type of Cells	S1P Receptor Expression	Major Effect	Reference
Neuroprogenitors/ Neurons	S1PR1–3,5	S1P/S1PR regulate neuroprogenitor proliferation, differentiation, and migration S1P receptor modulation by fingolimod phosphate increases BDNF expression in neurons.	[24,30,65,70] [87]
Astrocytes	S1PR1–3	S1P induces the gene expression and protein synthesis of *BDNF*, *HB-EGF*, *LIF*, *GDNF* and *PDGF-B*	[19,76,85,86]
Endothelial cells	S1PR1–3,4	S1P induces the gene expression and protein synthesis of *VEGF-C*	[19,48,81]
Ependymocytes	No data	Ependymocytes carry out S1P transport across BBB; ApoM-bound S1P contributes to the maintenance of ependymal cell polarity and CSF flow.	[31]
Oligodendrocyte Precursor Cells/ Oligodendrocytes	S1PR1–3,5	S1P regulates OPC survival, proliferation, and migration, regulates myelination	[18,19,45,49]

## Data Availability

No new data were created or analyzed in this study.

## Abbreviations

Akt	RAC-alpha serine/threonine-protein kinase (protein kinase B)
ASAH	acid ceramidase
BBB	Blood brain barrier
BDNF	brain-derived neurotrophic factor
c-FOS CERS	proto-oncogene, transcription factor, and key marker of neuronal activity ceramide synthases
CNS	central nervous system
CSF	cerebrospinal fluid
EGF	epidermal growth factor
ERK	extracellular signal-regulated kinase
EPCs	endothelial progenitor cells
FGF	fibroblast growth factor
FTY720	fingolimod
GFAP	glial fibrillary acidic protein
Gαi/o	αi/o subunit of G protein
Gαq	alpha subunit of G protein q
Gα12/13	alpha subunit of G protein family 12/13
GDNF	glial cell line-derived neurotrophic factor
HB-EGF	heparin-binding EGF-like growth factor
IGF	insulin-like growth factor
IL-1	interleukin-1
JNK	c-Jun N-terminal kinase
LIF	leukemia inhibitory factor
MAPK	mitogen-activated protein kinase
NGF	nerve growth factor
NT-3	neurotrophin 3
NT-4	neurotrophin 4
NT-4/5	neurotrophin 4/5
NTFs	neurotrophins
Nogo-A	a protein that restricts axon growth in the nervous system
p75NTR	p75 neurotrophin receptor
NSCs	neural stem cells
OPCs	oligodendrocyte precursor cells
OLDs	oligodendrocyte cells
PDGF-B	platelet-derived growth factor B
PI3K	phosphoinositide 3-kinase
Rho kinase	serine/threonine kinase, a key effector of small Rho A/B/C GTPases
S1P	sphingosine-1-phosphate
S1PR1–5	sphingosine-1-phosphate receptors 1–5
SGPL SGPP	S1P lyase sphingosine-1-phosphate phosphatase
SGZ	subgranular zone of the dentate gyrus
SK	sphingosine kinase
SLs	sphingolipids
SVZ	subventricular zone of the brain ventricles
tEAE	forebrain-targeted model of experimental autoimmune encephalomyelitis
TGF	transforming growth factor
TNF-α	tumor necrosis factor alpha
VEGF	vascular endothelial growth factor
